# Self-reported functional recovery after reconstruction versus repair in acute anterior cruciate ligament rupture (ROTOR): a randomized controlled clinical trial

**DOI:** 10.1186/s12891-018-2028-4

**Published:** 2018-04-20

**Authors:** Barbara C. Boer, Roy A. G. Hoogeslag, Reinoud W. Brouwer, Anna Demmer, Rianne M. H. A. Huis in ‘t Veld

**Affiliations:** 1 OCON Centre for Orthopaedic Surgery, Knee Unit, Hengelo, the Netherlands; 2 Department of Orthopaedic Surgery, Martini Hospital, Groningen, the Netherlands

**Keywords:** Anterior cruciate ligament (ACL), ACL reconstruction, ACL suture repair, Dynamic augmentation, Knee injury

## Abstract

**Background:**

Anterior cruciate ligament (ACL) reconstruction is today’s surgical gold standard for ACL rupture. Although it provides satisfactory results, not all patients return to their previous activity level and moreover, early posttraumatic osteoarthritis is not prevented. As such, a renewed interest has emerged in ACL suture repair combined with dynamic augmentation. Compared to ACL reconstruction, the hypothesized advantages of ACL suture repair are earlier return to sports, reduction of early posttraumatic osteoarthritis and preservation of the patient’s native ACL tissue and proprioceptive envelope of the knee. In recent literature, ACL suture repair combined with dynamic augmentation tends to be at least equally effective compared to ACL reconstruction, but no randomized comparative study has yet been conducted.

**Methods/design:**

This study is a prospective, stratified, block randomized controlled trial. Forty-eight patients with an ACL rupture will be assigned to either a suture repair group with dynamic augmentation and microfracture of the femoral notch, or an ACL reconstruction group with autologous semitendinosis graft and all-inside technique. The primary objective is to investigate the hypothesis that suture repair of a ruptured ACL results in at least equal effectiveness compared with an ACL reconstruction in terms of patient self-reported outcomes (IKDC 2000 subjective scale) 1 year postoperatively. Secondary objectives are to evaluate patient self-reported outcomes (IKDC 2000, KOOS, Tegner, VAS), re-rupture rate, rehabilitation time required for return to daily and sports activities, achieved levels of sports activity, clinimetrics (Rolimeter, LSI, Isoforce) and development of osteoarthritis, at short term (6 weeks, 3, 6 and 9 months and 1 year), midterm (2 and 5 years) and long term (10 years) postoperatively.

**Discussion:**

A renewed interest has emerged in ACL suture repair combined with dynamic augmentation in the treatment of ACL rupture. Recent cohort studies show good short- and midterm results for this technique. This randomized controlled trial has been designed to compare the outcome of suture repair of a ruptured ACL, combined with DIS as well as microfracture of the femoral notch, with ACL reconstruction using autologous semitendinosus.

**Trial registration:**

Clinical Trials Register NCT02310854 (retrospectively registered on December 1st, 2014).

## Background

Rupture of the anterior cruciate ligament (ACL) is one of the most common injuries of the knee [1]. Reported incidence varies between 0,3 and 0,8 per 1000 [2, 3] and most patients with ACL ruptures are young sportively active individuals (males 15–34 years; females 14–21 years) [4]. ACL rupture is a serious injury of the knee with high probability of the occurrence of dynamic instability, accompanying lesions and early post-traumatic osteoarthritis [5–10].

The treatment of ACL ruptures is aimed at achieving return to previous activity levels by resolving the instability of the affected knee, and preventing the development of post-traumatic osteoarthritis. The surgical gold standard is ACL reconstruction [11]. However, Biau et al. [12] concluded in a meta-analysis that only 40% of patients return to their previous activity levels after ACL reconstruction surgery. Moreover, the incidence of re-rupture of the reconstructed ACL is 3–22% within 2 years after surgery [13–15] and the risk of early posttraumatic osteoarthritis is still present [8–10].

In order to optimize the clinical results after ACL rupture, a renewed interest has emerged in ACL suture repair. In contemporary repair techniques, the sutured ACL is augmented with a strong, small diameter braid. In a biomechanical study, Kohl et al. demonstrated that, in contrast to static augmentation, dynamic augmentation is able to restore anterior-posterior stability of the knee directly postoperative as well as after cyclic loading [16]. In a pilot study, ACL suture repair combined with dynamic augmentation and microfracture in the femoral notch resulted in satisfactory clinical and radiological healing of the torn ACL at one and 5-year follow-up [17, 18]. Also, in three prospective cohort studies, two with one-year follow-up in 26 and 45 patients and one with 2 years follow-up in 69 (of 278) patients, ACL repair with dynamic augmentation provided successful functional recovery and patient self-reported outcomes [19–21]. Moreover, patients could return to their previous level of sports within 5 months after surgery [17, 20]. In terms of complications, the failure rate seems comparable to the re-rupture rate of ACL reconstruction. The pilot study of Eggli showed one failure in 10 patients after a 2 year follow-up and two failures in 10 patients after a 5 year follow-up [17, 18]. A larger cohort study of Henle showed 4% failure rate in 69 (of 278) patients after 2 years follow-up [20].

Ergo, ACL suture repair with dynamic augmentation seems to be a promising technique. However, to date no randomized comparative study has been conducted in which ACL suture repair with dynamic augmentation is compared with the gold surgical standard, ACL reconstruction. This study aims to investigate the hypothesis that suture repair of a ruptured ACL, combined with dynamic augmentation as well as microfracture of the femoral notch, will result in at least equal effectiveness compared with ACL reconstruction using autologous semitendinosus in terms of patient self-reported outcomes (IKDC 2000 subjective scale) 1 year postoperatively.

## Methods

### Study design

This study is a Medical Ethical Committee approved, (Medical Ethics Committee ‘Twente’, reference number NL50116.044.14/P1426) prospective, stratified and block randomized controlled trial: patients will be allocated to undergo either ACL suture repair or ACL reconstruction. The study will be conducted at the Centre for Orthopaedic Surgery OCON, Hengelo, The Netherlands.

**Fig. 1:**
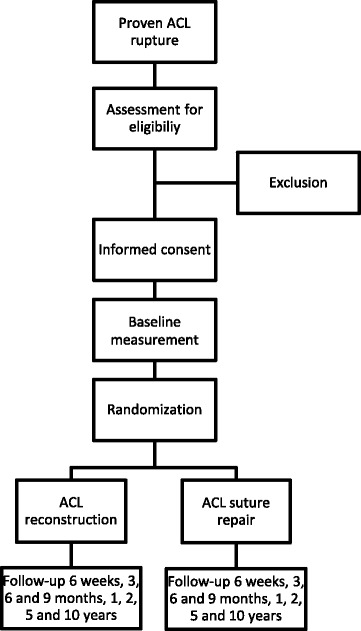
Flowchart of patient inclusion

Patients will be recruited at the outpatient department of OCON and informed about the study by their orthopaedic surgeon. After 2–3 days of reflection, an independent investigator will check eligibility and obtain informed consent. One orthopaedic surgeon will operate all patients. Blinding of the surgeon, physiotherapist who will conduct clinimetrics and patient is not used due to practical reasons. All patients will receive identical rehabilitation after surgery, apart from bracing in extension lock for the first 5 days postoperative in the repair group; the standard nationally used physiotherapy protocol regarding ACL reconstruction is given to the patient and their own physiotherapist. Patients will be followed-up in the outpatient clinic preoperatively as well as 6 weeks, 3, 6, 9 months, 1, 2, 5 and 10 years postoperatively (Fig. 1), where all the outcome measurements will be conducted. With weekly data management patients will be contacted in case of no show in order to minimize loss to follow-up. Protocol modifications will be communicated via amendments according to the guidelines of the Medical Ethical Committee.

### Study sample

Patients eligible for enrolment in this study are sportively active patients between 18 and 30 years with a proven primary ACL rupture, confirmed by means of history, physical examination and MRI, for whom an indication for ACL reconstruction surgery exists and who can undergo surgery within 21 days of injury. Exclusion criteria are concomitant large meniscal injury needing repair, cartilage injury requiring surgical intervention or ligamentous lesions of the ipsilateral knee, pre-injury Tegner score below 5, history of knee surgery of the ipsilateral knee, pre-existing significant malalignment of the ipsilateral knee, hypersensitivity to cobalt, chromium or nickel, muscular, neurological or vascular abnormalities, osteoarthritis, use of prednisone or cytostatics, tendency to form excessive scar tissue, pregnancy, osteoporosis or infection.

Patients can withdraw from the study at any time. They will receive appropriate treatment according to standard-care.

### Intervention

#### ACL suture repair

ACL suture repair will be performed within 21 days after injury. The dynamic intraligamentary stabilisation (DIS) technique will be used (Ligamys, Mathys Medical, Bettlach, Switzerland). The procedure is started with standard arthroscopy to assess all compartments for concomitant injury. When a patient meets one of the exclusion criteria, they will be excluded from the study. Patients will receive further standard care including ACL reconstruction after meeting the Millet criteria. Otherwise, the procedure will be continued and the ACL rupture type will be classified. The tibial attachment of the ACL is identified using an intra-articular guide. A guide wire is overdrilled in the metaphysis and the monoblock is screwed in place. A suture to secure the ACL remnant is inserted through the screw and passed through the ligament and pulled through the femur. The femoral attachment is then marked using a guide from the anteromedial portal. A polyethylene wire will be pulled distally through the femur towards proximal tibia. The wire is stabilized at the femoral position with a cortical suspension button. The polyethylene wire is pulled through the tibia and tightened before final tension is applied. The procedure will be completed by microfracture of the femoral attachment.

Removal of the Ligamys spring will take place in day care, no earlier than after recording of the primary outcome measure 1 year after surgery. The previous anteromedial incision will be used.

#### ACL reconstruction

ACL reconstruction will be performed when the patient meets the Millett criteria [22], usually approximately 6 weeks after injury. If necessary, additional physiotherapy will be given and patients will be rescheduled for a retest. In that case, the measurements of the retest where the Millet criteria are met will be used as baseline measurement during the study. An all-inside technique (Arthrex, Naples, Florida, USA) will be used. The semitendinosus tendon from the ipsilateral leg will be harvested and quadrupled. A standard arthroscopy will be performed for diagnosis and treatment of concomitant injuries and evaluation of ACL rupture morphology. When a patient meets one of the exclusion criteria, he will be excluded from the study, but the surgical procedure will be continued. ACL rupture will be classified. After removing ACL remnants, the tibial and femoral tunnel will be prepared. The graft will be positioned in the femoral tunnel first and fixed with a cortical suspension button with variable loop length. After, the graft will be placed in the tibial tunnel and fixed with a cortical suspension button, with the knee in 0 degrees of extension while anterior translation of tibia in relation to femur is eliminated. Positioning and tension of the graft will be verified under vision, and if necessary, the graft will be tightened.

### Main study parameter/endpoint

The primary objective of this non-inferiority study is to determine whether ACL repair will result in at least equal effectiveness compared with ACL reconstruction in terms of the self-reported functional outcome measured by the International Knee Documentation Score 2000 subjective knee evaluation score (IKDC Subjective) 1 year postoperative (Table 1) [23–25].

**Table 1:** Oversight of the investigations and follow-up moments

	B	OR	6w	3 m	6 m	9 m	1y	2y	5y	10y
X-ray	x	x					x	x	x	x
MRI	x									
IKDC 2000 Subjective	x		x	x	x	x	x	x	x	x
IKDC 2000 Current health	x	x	x	x	x	x	x	x	x	x
IKDC 2000 History	x	x	x	x	x	x	x	x	x	x
IKDC 2000 Demographic form	x	x	x	x	x	x	x	x	x	x
IKDC Physical examination	x		x	x	x	x	x	x	x	x
KOOS	x		x	x	x	x	x	x	x	x
Tegner score	x		x	x	x	x	x	x	x	x
VAS satisfaction	x		x	x	x	x	x	x	x	x
AP laxity	x		x	x	x	x	x	x	x	x
LSI power tests	x				x	x	x	x	x	x
LSI jump tests					x	x	x	x	x	x
Sport specific fatigue test							x			
Concomitant injury		x								
Rupture pattern		x								
Quality repair		x								
Complications & side effects		x	x	x	x	x	x	x	x	x
Re-rupture			x	x	x	x	x	x	x	x

*B* baseline, *OR* peri-operative, *w* weeks, *m* months, *y* years, *IKDC* International Knee Documentation Score, *KOOS* Knee Injury and Osteoarthritis Outcome Score, *VAS* Visual Analog Scale, *AP* anteroposterior, *LSI* leg symmetry index

### Secondary study parameters

Secondary outcomes of this study are: to determine any between group differences in self-reported functional outcomes, clinimetrics and development of osteoarthritis at 6 week, 3, 6 and 9 months, 2, 5 and 10 years after surgery and secondly, whether between groups differences exist, both with respect to perioperative classification of the ACL rupture type and onset of failure of the ACL repair or reconstruction.

Differences in baseline characteristics will be recorded by the use of IKDC 2000 demographic form, IKDC 2000 Current Health, IKDC 2000 History and reported complications and side effects.

Patient self reported outcomes will assess the patients’ perceived level of functional recovery (IKDC Subjective Scale) [23–25], daily life activities (Knee Injury and Osteoarthritis Outcome Score (KOOS)) [26], level of physical activity (Tegner Activity Level) [27, 28], knee pain (Visual Analogue Scale (VAS)) and satisfaction with the outcome of surgery. Clinimetrics will be assessed by the IKDC 2000 physical examination score [23–25], including instrumented anteroposterior laxity (Rolimeter) [29–32] as well as leg symmetry index (LSI) for Gustavsson’s jump test battery and isokinetic quadriceps and hamstrings force measured by a dynamometer (Isoforce) [33].

Additional secondary outcomes are knee kinematic parameters (i.e. degree of flexion and varus/valgus angles) 1 and 2 year(s) after surgery; the jump tests will be instrumented and patients will be equipped with inertial sensors (Xsens Technologies) for these tests. Furthermore, in order to explore the role of long lasting exertion (one-hour running and pivoting protocol) on neuromuscular fatigue and knee kinematics, a sport-specific fatigue test with EMG measurement of quadriceps and hamstring activity, and inertial biomechanical sensors to measure functional biomechanical parameters (Xsens Technologies) will be performed in a subgroup 1 year after ACL suture repair.

Two independent radiologists will evaluate the Kellgren and Lawrence score for radiologic signs of osteoarthritis on anteroposterior and lateral weight baring X-rays [34].

Failure, defined as the occurrence pathological laxity or subjective instability, or the discontinuity of the ACL suture repair or reconstruction based on MRI or arthroscopy, as well as other complications will be recorded. Also, perioperative classification of ACL rupture type (tear location (proximal, midsubstance, distal rupture), rupture pattern (single strand, two bundles, three or more strands), and synovial sheath (completely intact, > 50% intact, < 50% intact)) [20] as well as perioperative classification regarding quality of the repair (anatomical, nearly-anatomical, non-anatomical) will be assessed [35].

### Randomization

After inclusion, patients will be randomized into an experimental group (repair) or a control group (reconstruction), in blocks with varying sizes (*N* = 2 and *N* = 4) by an independent investigator with PASS (Power Analyses and Sample Size Software; rand.exe version 6.0). The extent of physical activity in daily life poses a potential risk to repair/graft failure. To make sure both groups have an equal risk of failure, patients will be stratified based on physical activity level using the Tegner score (moderate: Tegner 5-7; high: Tegner 8-10) [27, 28].

### Sample size determination

In order to detect non-inferiority of ACL repair compared to ACL reconstruction surgery in terms of patient self-reported functional outcome measured by the IKDC Subjective score, to achieve a power of 90% and an alpha of 5%, a sample size of 20 patients in each study group is required. According to literature, it seems relevant to consider a standard deviation at nine in both groups [36]. A difference of 11, 5 points in score of IKDC 2000 is suggested as clinically relevant [37]. The margin of equivalence 10 lies within this clinically relevant effect size.

Considering a lost-to-follow-up rate of 20%, it is planned to include 24 patients per randomization group. Thus, in total 48 patients will be included in the study.

### Statistics

The identified data will be entered into and analysed with SPSS version 23 (IBM SPSS, Chicago, Illinois, USA) by the trialcoordinator who is not involved in data collection and therefore not blinded to group allocation of participants. Trial results will be published in scientific journals.

#### Descriptive statistics

Baseline characteristics will be presented as mean ± SD or median (range) for continuous data and as numbers with corresponding percentages for categorical data as appropriate. Comparisons between randomized groups will be analysed using X^2^ or Fisher’s exact test for categorical variables and the Student T-test or Mann-Whitney U test for continuous variables, with normality verified by the Shapiro-Wilk test and histograms.

#### Primary study parameter

Differences between IKDC 2000 at baseline and one-year post-operative will be determined for each group, as well as differences between the groups at each follow-up. In case of clinically meaningful differences between groups (> 10 points on the IKDC2000), the non-inferiority hypothesis will be rejected. In case of non-inferiority, superiority analyses will be conducted. For normally distributed data mixed models analyses for repeated measures will be used. In case of non-normally distributed data, the Friedman test for differences within groups, and Mann-Whitney Y test for differences between groups, will be used. A distinction will be made between short term (6 weeks, 3, 6, 9 months and 1 year), mid-term (2 and 5 year) and long-term (10 year) postoperative outcomes.

#### Secondary study parameters

For IKDC 2000, KOOS, Tegner, VAS, IKDC 2000 physical examination, AP-laxity, LSI for jump tests, isokinetic quadriceps and hamstring force as well as re-rupture, the same statistics as described above will be used. Re-rupture of 10% or more is considered to be clinically relevant. For the sport-specific fatigue protocol repeated measures ANOVA with SIDAK t-test post hoc test will be performed.

## Discussion

This paper reports on the study design of the ROTOR (RecOnsTruction Or Repair) trial, which will compare the subjective, objective and functional outcomes of hamstring autograft, the current gold-standard surgery for ACL rupture, with those for suture repair while following the participants from short to long term. The conduct of this study is important, since there is growing evidence that suture repair of the ruptured ACL augmented with a dynamic joint bridging stabilisation technique combined with micro fracturing in the femoral notch leads to good short and midterm results [16–21]. The hypothesis of this study is that suture repair will result in at least as satisfactory outcomes as ACL reconstruction. A proposed mechanism for this thesis is the retention of the patient’s ACL resulting in healing of ligament tissue, the restitution of native ligament proprioception and restoration of postero-anterior laxity, whereas reconstruction with the process of ligamentization of harvested tendon provides biomechanical stability only.

However, despite several prospective cohort studies, no high quality randomized controlled trial (RCT) comparing ACL suture repair with dynamic augmentation and ACL reconstruction have been published so far. In this RCT a broad range of parameters will be evaluated, including patient self-reported outcomes (IKDC 2000, KOOS, Tegner, VAS), re-rupture rate, rehabilitation time required for return to daily and sports activities, achieved levels of sports activity, clinimetrics (Rolimeter, LSI, Isoforce) and development of osteoarthritis, at short term, midterm and long term postoperatively. The use of intertial sensors for jump tests may provide insight in increased neuromuscular knee control, which is considered an advantage of ACL suture repair due to retaining of the proprioceptive function, compared to ACL reconstruction. Follow-up will take place at short term, midterm and long term postoperative.

To our knowledge, this is the first randomized controlled trial to investigate the functional recovery after ACL suture repair in comparison to ACL reconstruction. This trial has the potential to demonstrate, with a good level of evidence, the effectiveness of ACL suture repair combined with dynamic augmentation as well as microfracture of the femoral notch compared to the gold standard ACL reconstruction using autologous hamstring. As it is not yet clarified what the exact indications for this procedure are and in order to minimize the possible influence of confounding factors on the effectiveness of repair or reconstruction, patients suffering from severe common concomitant injury in ACL rupture, i.e. large meniscal injury needing repair or cartilage injury requiring surgical intervention, were excluded in the current study. Despite the fact that it might limit the generalisability of our findings, it does provide a higher intrinsic validity. If proven at least equally effective, ACL suture repair with dynamic joint bridging stabilisation can be considered as an innovative treatment for ACL ruptures.

## Abbreviations

ACL: Anterior cruciate ligament
DIS: Dynamic intraligamentary stabilization
IKDC: International knee documentation score
KOOS: Knee injury and osteoarthritis outcome score
LSI: Leg symmetry index
PASS: Power Analyses and Sample Size Software
RCT: Randomized controlled trial
VAS: Visual analog scale
